# Control of Seed Germination and Plant Development by Carbon and Nitrogen Availability

**DOI:** 10.3389/fpls.2015.01023

**Published:** 2015-11-18

**Authors:** Daniel Osuna, Pilar Prieto, Miguel Aguilar

**Affiliations:** ^1^Institute for Sustainable Agriculture, Agencia Estatal Consejo Superior de Investigaciones Científicas, Córdoba, Spain,; ^2^Área de Fisiología Vegetal, Facultad de Ciencias, Universidad de Córdoba, Córdoba, Spain

**Keywords:** carbon, nitrogen, nitric oxide, abscisic acid, auxin, gibberellin, cytokinin

## Abstract

Little is known about the molecular basis of the influence of external carbon/nitrogen (C/N) ratio and other abiotic factors on phytohormones regulation during seed germination and plant developmental processes, and the identification of elements that participate in this response is essential to understand plant nutrient perception and signaling. Sugars (sucrose, glucose) and nitrate not only act as nutrients but also as signaling molecules in plant development. A connection between changes in auxin transport and nitrate signal transduction has been reported in Arabidopsis thaliana through the NRT1.1, a nitrate sensor and transporter that also functions as a repressor of lateral root growth under low concentrations of nitrate by promoting auxin transport. Nitrate inhibits the elongation of lateral roots, but this effect is significantly reduced in abscisic acid (ABA)-insensitive mutants, what suggests that ABA might mediate the inhibition of lateral root elongation by nitrate. Gibberellin (GA) biosynthesis has been also related to nitrate level in seed germination and its requirement is determined by embryonic ABA. These mechanisms connect nutrients and hormones signaling during seed germination and plant development. Thus, the genetic identification of the molecular components involved in nutrients-dependent pathways would help to elucidate the potential crosstalk between nutrients, nitric oxide (NO) and phytohormones (ABA, auxins and GAs) in seed germination and plant development. In this review we focus on changes in C and N levels and how they control seed germination and plant developmental processes through the interaction with other plant growth regulators, such as phytohormones.

## Introduction

A seed needs to integrate all the signals that represent its nutritional status in order to achieve germination when the appropriate nutrients conditions are present. Only when the right hormonal response takes place, seed germination is induced and seedlings become mature plants.

Nutrients are also known to influence seedling development and some processes may be mediated by the absolute levels of a particular sugar, such as glucose (Borisjuk et al., 1998) or sucrose (Borisjuk et al., 2002), whereas other processes may be related to metabolic events associated with the presence of carbohydrates at high concentrations, rather than the presence of high concentrations of carbohydrate *per se* (Krapp et al., 1993). Moreover, sugar-responsive pathways exhibit crosstalk with nitrogen-responsive pathways (Coruzzi and Bush, 2001; Coruzzi and Zhou, 2001). Therefore, C/N balance seems to be crucial for the regulation of gene expression by carbohydrates and nitrogen (Lejay et al., 1999; Oliveira and Coruzzi, 1999; Zhuo et al., 1999; Zheng, 2009). In spite of the importance of C/N balance signaling, the number of genes so far implicated in this process is scarce. Among these genes, *NRT2.1* and *NRT2.2* are high-affinity nitrate transporters in roots, particularly important under non-limiting nitrogen growth conditions (Orsel et al., 2002). *GLR1.1*, a putative glutamate receptor 1.1, connects carbon and nitrogen metabolism, ABA metabolism and water stress response in *Arabidopsis* (Kang et al., 2004). *OSU1/QUA2/TSD2*-encoded putative methyltransferase may act in cell wall biogenesis (Mouille et al., 2007) and is necessary for a normal response to C/N balance (Gao et al., 2008). Different targets of OSU1/QUA2/TSD2 would regulate pectin biosynthesis and responses to C/N balance, thus connecting cell wall biogenesis and C/N balance response (Zheng, 2009). OSU1 seems to work by down-modulating different pathways resulting in high or low C/N responses. Alternatively it could work on a single pathway regulating several transcription factors in response to C/N imbalance conditions (Zheng, 2009). Finally, it has been demonstrated the involvement of specific ABA and SnRK1s signaling pathways in C/N response under ABI1 regulation (Lu et al., 2015). Therefore, for each identified molecular player it is essential to check whether there is a signal representing C/N balance signal or the C/N balance sensing network just results from the crosstalk between carbohydrates and nitrogen pathways.

Genes involved in metabolism, protein synthesis and degradation, RNA metabolism, signal transduction and hormones (auxin, gibberellin, cytokinin, ethylene, abscisic acid and brassinosteroids) pathways may play important roles in the C/N balance or ratio response, and nutrients crosstalk has also been reported (Palenchar et al., 2004; Price et al., 2004; Scheible et al., 2004; Gutiérrez et al., 2007; Osuna et al., 2007). Additionally, C/N signaling systems are subject to a “matrix effect” in which downstream responses are dependent upon cell-type plus developmental, metabolic, and/or environmental conditions (Coruzzi and Zhou, 2001).

This review is focused on the mechanisms underlying the regulation of seed dormancy, seed germination and seedling development by carbon and nitrogen nutrient balance. Specific C- and N-regulatory pathways and C/N interaction pathways controlling these developmental processes will be described and discussed.

## N-Control of Development

### Seed Dormancy and Germination

Germination is classically described as a triphasic process determined by water relations. Phase I: the seed imbibes and resumes metabolism. Phase II: water uptake by the seed reaches a plateau and stays in a dormant state. Phase III: water uptake is resumed and the radicle emerges from the seed (Finch-Savage and Leubner-Metzger, 2006).

The depth of seed dormancy was inversely correlated to seed nitrate content, “endogenous nitrate” (Alboresi et al., 2005). Production of dormant seeds was inhibited in plants grown in high concentration of nitrate (50 mM) during seed maturation when compared to plants grown under standard conditions (10 mM nitrate). The *nia1/nia2* double mutant, which accumulates nitrate under standard conditions, produces seeds that are less dormant than WT seeds under identical conditions (Wilkinson and Crawford, 1993). Nitrate uptake by NRT1.1 was suggested to play a relevant role in dormancy regulation (Alboresi et al., 2005). One of the genes encoding nitrate reductase, *NR1*, was highly expressed under conditions that break dormancy, including cold, light and nitrate (Finch-Savage et al., 2007). Unlike *NR1*, expression of *NiR* gene may depend on nitrate. However, dormancy release was not accompanied by increased transcript abundance of none of the seven (putative) glutamine synthetase (*GS1*) genes. Control of nitrate assimilation at the (post) translational level was also analyzed, and the nitrate reductase mutant G′4-3 of *Arabidopsis*, with just 0.5% of the nitrate reductase activity of the corresponding wild type, was even less dormant than the wild type (Alboresi et al., 2005). Thus nitrate uptake and reduction to nitrite seems to generate a signal for dormancy breaking instead of just being a source of an essential nutrient.

**FIGURE 1:**
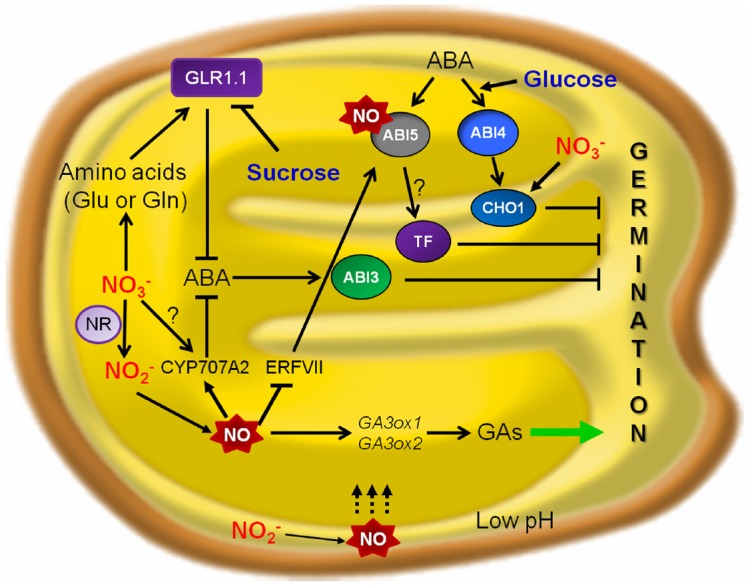
**A hypothesized model for nutrients signaling crosstalk with NO and ABA during seed germination.** The figure highlights the action points of nitrate and glucose in NO and ABA signaling pathways controlling alleviation of seed dormancy and promotion of *Arabidopsis* seed germination. In the context of ABA-mediated inhibition of seedling establishment, NIA/NR- and AtNOA1-mediated pathways of NO biosynthesis function in an additive way. NO-deficient seedlings showed a higher basal and induced expression of ABA-responsive genes, conferring enhanced resistance to dehydration. In *Arabidopsis*, ABA 8′-hydroxylase encoded by *CYP707A2* is implicated in NO-mediated ABA catabolism and seed dormancy break (Liu et al., 2009). In the seed endosperm, group VII ERFs act as NO sensors and regulate *ABI5* (*ABSCISIC ACID INSENSITIVE 5*), highlighting as molecular players in NO-ABA crosstalk during seed germination (Gibbs et al., 2014). NO nitrosylates ABI5 as a regulator to control ABA hormone signaling through its degradation (Albertos et al., 2015). AtGLR1.1 is a component of the HXK1-independent ABA pathway and can be expressed in presence of a N source such as nitrate or amino acids (Glu or Gln), promoting seed germination (Kang and Turano, 2003; Kang et al., 2004). NR, nitrate reductase; NO, nitric oxide; ABA, abscisic acid; GAs, gibberellins; *GA3ox1* and *GA3ox2*, Gibberellic acid oxidase1 and 2; *CYP707A2*, Cytochrome P450 ABA 8′-hydroxylase; GLR1.1, putative glutamate receptor 1.1; TF, transcription factor.

Nitrate can enhance ABA catabolism and inhibit ABA synthesis (Ali-Rachedi et al., 2004). Addition of nitrate to dormant seeds resulted in less ABA content and supress *de novo* synthesis. Seed dormancy was broken by nitrate, nitrite and CN^-^ through NO production and altered ABA sensitivity (Bethke et al., 2006a). As shown by Matakiadis et al. (2008), regulation of ABA degradation is more important than regulation of ABA synthesis for the control of seed dormancy by exogenous nitrate. They proposed that *CYP707A2*, a gene involved in ABA catabolism, could play a role in the control of seed ABA content by endogenous nitrate (Figure 1). In addition, other genes like *NCED9* must be involved in the control of dormancy by nitrate, since *cyp707a2* mutant seeds behaved as the wild-type under 10 mM nitrate.

In *Arabidopsis*, there are two genes encoding nitrate reductase (NR), *NIA1* and *NIA2*. NIA1, which is expressed at much lower level than NIA2, seems to be responsible for NO production in the context of ABA signaling (Bright et al., 2006). Recently, Lozano-Juste and León (2010) have demonstrated that NO production in *Arabidopsis* is mostly associated to NO biosynthesis mediated by NIA/NR and AtNOA1 (Nitric Oxide-Associated1). Seed dormancy, hypersensitivity to ABA during seed germination and establishment, as well as dehydration resistance, all showed a good correlation with an increasing deficiency in NO of *nia1nia2*, *noa1-2*, and *nia1nia2noa1-2* plants.

In *Arabidopsis* and barley, NO seems to be an endogenous regulator of seed germination (Figure 1), since it can disrupt seed dormancy (Bethke et al., 2004b, 2006a,b; Libourel et al., 2006). A similar function for NO was suggested by Sarath et al. (2005) in warm-season C_4_-grasses. Switchgrass seeds responded positively to NO, and NO could disrupt the residual dormancy found in cold-stratified switchgrass seeds.

NO or KCN vapors induced germination in seeds lacking a testa, suggesting that NO was perceived in the aleurone cells or in the embryo. Aleurone layer perceived NO and responded to it because vacuolation was inhibited by the NO scavenger (Bethke et al., 2007), strongly suggesting that *Arabidopsis* aleurone cells synthesize NO. There are no evidences that NO is produced enzymatically. On the contrary, NO seems to be generated in the apoplast of the aleurone cell layer by non-enzymatic reduction of nitrite at acidic pH in germinating seeds, and catechin would also stimulate the production of NO from nitrite with a maximum at pH 3–4 (Bethke et al., 2004a,b). Nitrite either entering the grain from soil solution or released by the embryo axis, the scutellum, or the aleurone layer to the apoplast/endosperm cavity, would result in NO production (Bethke et al., 2004a), and apoplast of both GA- and ABA-treated *Hordeum vulgare* aleurone layers can produce NO from nitrite. NO would be an ideal signal for coordinating the activities of the embryo axis, scutellum, and aleurone layer in real time. Removal or damage of the aleurone layer resulted in embryo growth, demonstrating that one function of the *Arabidopsis* aleurone layer is to maintain the dormancy of imbibed seeds.

In *Arabidopsis thaliana* seeds, sodium nitro-prusside, cyanide, nitrate and nitrite decreased dormancy and the NO scavenger c-PTIO effectively promoted the maintenance of seed dormancy (Bethke et al., 2006b). NO was required to complete dormancy loss initiated by nitrate or nitrite, and cPTIO maintained dormancy in nitrite- and nitrate-treated seeds. Experimental data from Libourel et al. (2006) showed that exogenous gaseous NO is sufficient to disrupt seed dormancy. Cadman et al. (2006) have also suggested that, in *Arabidopsis*, the transcriptome anticipates growth initiation during dormancy-breaking. An increased *NR1* transcript abundance would anticipate nitrate assimilation during seedling growth. In switchgrass, however, seed germination was not significantly stimulated by nitrite or nitrate alone, and ferrocyanide was more effective than cyanide (Sarath et al., 2005).

GAs promote germination (Yamaguchi and Kamiya, 2002) and GA deficient mutants fail to germinate (Ross et al., 1997). The importance of ABA/GA balance for germination is supported by the fact that the reduced dormancy of some of the ABA mutants was linked to a lower GA-requirement for germination (Koornneef et al., 2002). GAs promote rapid degradation of DELLA proteins (intracellular repressors of GA responses), which leads to activation of the signaling pathway, and the comparison of gene expression in *ga1-3*. In DELLA-domain protein-deficient mutants (*gai*, *rga*, *rgl1*, and *rgl2*) in the *ga1-3* background allows to suggest the existence of DELLA-dependent and –independent gene expression pathways during germination (Cao et al., 2006). NO is also necessary for the transcription of *GA3ox1* and *GA3ox2* genes, involved in the biosynthesis of GA, which is needed for the vacuolation of cells in isolated aleurone layers in the absence of NO (Bethke et al., 2007). This suggests the possibility that NO could coordinate the reduction of dormancy imposed by ABA with the initiation of germination stimulated by GA (Figure 1). Hydroxylamines, nitrites, and other nitrogenous compounds yielding NO under strong oxidation are the striking promotors for germination of some kinds of seeds (Hendricks and Taylorson, 1974).

### Root and Shoot Development

Plants have mechanisms for sensing nitrate as a signal for inorganic-N status, whereas metabolites derived from nitrate may serve as signals for organic-N status. It is also known than nitrogen sensing regulates phenotypic changes in plants. Nitrateactivated transcription of a set of genes is related to the positive regulation of nitrate transport, whereas ammonium or glutamine have been proposed to negatively regulate this process (Touraine, 2004). A root-specific *ANR1* gene (a member of the MADS box transcription factors family), is nitrate-induced and appears to control lateral root growth (Zhang and Forde, 1998). There are examples of control by nitrogen both at the transcriptional and post-transcriptional level, and also examples of the matrix effect (Coruzzi and Zhou, 2001).

In a nitrate re-addition experiment to *Arabidopsis* liquid cultures transcriptional reprogramming of hormone metabolism and sensing was identified as one of the early responses to nitrate addition (Scheible et al., 2004). Several genes involved in CK synthesis, including *IPT3* and several downstream response elements involved in CK signaling, were induced around 30 min after adding 3 mM nitrate (KNO_3_), and other genes associated with CK signaling were up-regulated after 3 h. *DF4*, a gene involved in brassinosteroid biosynthesis and other genes involved in GA synthesis were also up-regulated after 3 h. Many 1-aminocyclopropane-1-carboxylate oxidases and other genes involved in ethylene synthesis and sensing were repressed.

ABA-insensitive mutants (*abi4-1*, *abi4-2*, and *abi5*) suffer a reduction of the systemic inhibitory effect of nitrate (Signora et al., 2001). Among other features of their phenotype, they show an increased elongation of lateral root at high concentrations of nitrate, what suggests that ABA is an important mediator of the inhibitory effect of nitrate on lateral root elongation. In *Arabidopsis*, nitric oxide (NO) can reduce primary root growth and also induce lateral root development, both processes being also regulated by auxins. In *A. thaliana* there is also an anthocyanin biosynthesis pathway that is specifically induced by nitrate limitation, in which pathway NLA (Nitrogen Limitation Adaptation) is an essential molecular component (Peng et al., 2008).

**FIGURE 2:**
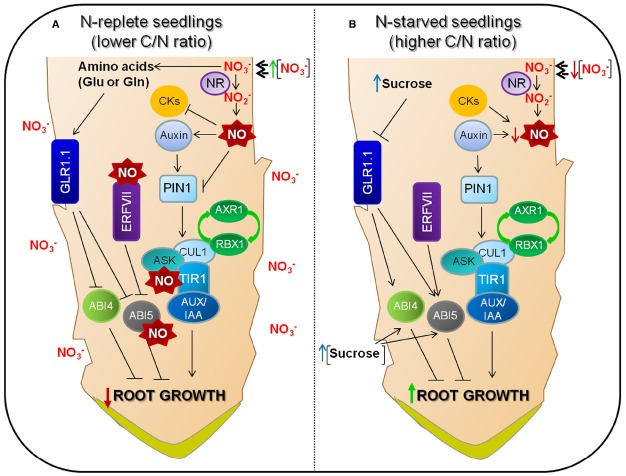
**Molecular components of nitrate signaling networks and their involvement in root development.** There is a transport-mediated “reflux” of auxin from the lateral root cap through the epidermis to the basal meristem and then back toward the root tip (Overvoorde et al., 2010). **(A)** Nitrate is reduced to nitrite by root nitrate reductase (NR). NO biosynthesis, mediated by NIA/NR- and AtNOA1 (Nitric Oxide-Associated1), is active and responsible for most of the NO production in *Arabidopsis* (Lozano-Juste and León, 2010). NO inhibits IAA oxidase, so reducing auxin degradation (Xu et al., 2010). However, increased NO levels have an interfering effect on acropetal auxin transport through PIN1 auxin efflux carrier, which shows a good correlation with a reduction in auxin response (Fernández-Marcos et al., 2011; Sanz et al., 2015). NO also has a positive effect on auxin signaling by *S*-nitrosylation of the auxin receptor F-box protein TIR1 (Terrile et al., 2012). ABI4, probably controls PIN1 repression through ABI3 on lateral root development (Wind et al., 2013). The group VII ERF transcription factors are degradated through the N-end rule pathway and are related to NO sensing, regulating the expression of *ABI5* (Gibbs et al., 2014). **(B)** Under low nitrate conditions, because of reduced NO levels, the auxin that is generated in the root n contributes to keep the gradients and maxima that are required to enhance root development (Ljung et al., 2005; Ikeda et al., 2009; Petersson et al., 2009). GLR1.1 is a molecular player and could regulate ABA biosynthesis and signaling to control root growth. Arrows and bars mean positive and negative effects, respectively.

Auxins are essential for plant root development, and both shoot- and root-synthesized auxins contribute to root organization and development. Shoot-derived auxins move to root cells by diffusion, a mechanism that differs from the mechanism of directing auxin distribution through polar auxin transport (PAT) occurring in roots. The same pathway that is followed by carbohydrates from “source to sink” also enables the long-distance flow of auxin and other hormones such as ABA and cytokinins (Robert and Friml, 2009). A good number of genes involved in auxin biosynthesis are expressed in roots, and the auxin that is generated in roots is partly responsible for the gradients that are necessary for normal root development (Ljung et al., 2005; Ikeda et al., 2009; Petersson et al., 2009). Auxin signaling comprises a number of molecular components and works by stabilizing the interaction of the TRANSPORT INHIBITOR RESPONSE 1 (TIR1) protein or the AUXIN FBOX PROTEINs (AFBs) with proteins of the Aux/IAA family (Overvoorde et al., 2010; see Figure 2). TIR1 and AFB proteins contribute to substrate specificity for the E3 ubiquitin-ligase activity of Skp1-Cul1-F-box (SCF) complexes (Chapman and Estelle, 2009). The IAA-stabilized interaction of SCF (TIR or AFB) enables Aux/IAA ubiquitination and the action of the 26S proteasome to remove these proteins (Maraschin Fdos et al., 2009).

Nitric oxide treatment affects meristem size in the primary root mainly by decreasing cell-division rates and promoting cell differentiation (Figure 2; Fernández-Marcos et al., 2011). A reduction in meristem size was also observed in the NO overaccumulating mutant chlorophyll a/b binding protein underexpressed 1/NO overproducer 1 (*cue1/nox1*). Interestingly, the root apical auxin maximum is altered after NO addition. Auxin transport and the level of the auxin efflux protein PINFORMED 1 (PIN1) are reduced significantly in the *cue1/nox1* double mutant. The abnormal organization of the quiescent center and surrounding cells of *cue1/nox1* double mutants resembles the phenotype of *pin1*-mutant roots, what suggests a connection between NO and auxin signaling in maintaining the activity and the size of the root apical meristem (Fernández-Marcos et al., 2011).

Changes in NR levels during plant growth modify NO levels and would link metabolism to gene regulation, and NO derived from NR activity modulates group VII ERF transcriptional regulators stability through targeted proteolysis (N-End Rule Pathway in plants; Gibbs et al., 2014). Still it is necessary to check if, similarly to seed germination, group VII ERFs regulate NO/ABA signaling by controlling the expression of *ABI5 (ABA INSENSITIVE 5*) in seedlings grown under different C/N regimes.

Nitrogen nutrition has significant effects on root and shoot relations (Feng and Liu, 1996; Lioert et al., 1999). Nitrogen deficiency increased root surface area, increased consumption of assimilates, reduced the amount of nitrogen translocated to shoot, diminished shoot growth, and yielded an increased root/shoot ratio. Extra nitrogen nutrition, however, caused a shoot overgrowth, reduced the amount of assimilates availability for root, and decreased the root/shoot ratio (Passioura, 1983).

All in all, root growth regulation could be summarized as follows: auxin flows from the lateral root cap to the basal meristem and returns to the root tip (Overvoorde et al., 2010). Nitrate is reduced to nitrite by nitrate reductase in the root. NIA/NR- and AtNOA1 (Nitric Oxide-Associated1)-mediated synthesis of NO is active and generates most of the NO in *Arabidopsis* (Lozano-Juste and León, 2010). NO maintains auxin levels by inhibiting IAA oxidase activity (Xu et al., 2010), but high NO interfere with acropetal auxin transport through PIN1 auxin efflux carrier, which correlates well with a reduced response to auxin (Fernández-Marcos et al., 2011; Sanz et al., 2015). NO also has a positive effect on auxin signaling through *S*-nitrosylation of TIR1 (Terrile et al., 2012). In N-replete seedlings (lower C/N ratio condition), increased NO levels would nitrosylate ABI5 as a regulator to control ABA hormone signaling through its degradation (Albertos et al., 2015). On the other hand, ABI5 is stabilized through its phosphorylation upon ABA treatment becoming active (Hu et al., 2014). Therefore, characterization of ABI5 post-translational modifications is relevant, and it bound to NO interference on acropetal auxin transport through PIN1 auxin efflux carrier, establishes a limited root development (Figure 2A). Under low nitrate conditions, because of reduced NO levels, the auxin that is generated in the root contributes to maintain the gradients and maxima required for enhanced root development (Ljung et al., 2005; Ikeda et al., 2009; Petersson et al., 2009). Additionally, in N-starved seedlings, auxin and cytokinins (CKs) could increase NO production to basal levels, similar to other stresses such as iron deficiency (Chen et al., 2010). CKs are known to induce NO biosynthesis depending on plant cell status (Yu et al., 1998), and auxins could increase NO production similarly to other stresses such as iron deficiency (Chen et al., 2010). These minimum NO levels would not be enough to repress PIN1 expression, not altering acropetal auxin transport, therefore promoting enhanced root growth (Figure 2B). It seems that low NO levels generated in N-starved root seedlings (higher C/N ratio) are determinants for enhanced lateral root growth rate (Figure 2B). Moreover, ABI5 post-translational state could regulate their downstream transcription factors in an exquisite way, causing an effect in metabolism, growth and plant development.

## C-Control of Development

### Seed Dormancy and Germination

In *Arabidopsis* seeds, dormancy release can be activated by cold, nitrate and light. The efficiency of this activation depends on the extension of the dry after-ripening period following harvest (Finch-Savage et al., 2007). Light is essential for germination, though dormancy is not released by light if seeds have not been subject to an extended period of after-ripening or a combination of a shorter period of after-ripening plus imbibition on a nitrate solution or cold treatment.

Regulation of seed germination under an excess of nutrient supply has also been deeply studied. *CHO1* codes for a putative transcription factor with two AP2 domains, expressed predominantly in seed, with the strongest expression 24 h after seed imbibition. Under an excess of glucose an nitrate supply, ABA signaling pathway could be influenced at different levels, and transcription factor CHOTTO1 (a double APETALA2 domain protein of *Arabidopsis thaliana*) is responsible for germination arrest (Yamagishi et al., 2009). *Arabidopsis cho1* mutants are resistant to the ABA analog (–)-R-ABA during seed germination and seedling development (Yamagishi et al., 2009). Only weak resistance to exogenously applied (+)-S-ABA was observed during seed germination of *cho1* seeds, and *abi4 cho1* double mutant seeds germinated at rates comparable to those of the *abi4-5* mutant seeds in the presence of exogenous (+)-S-ABA. Moreover, the induction of *CHO1* expression did not occur in the *abi4* mutants, suggesting that CHO1 acts in the same pathway as ABI4. ABI4 is involved in glucose signaling, and therefore operates the downstream target CHO1. As a result, both *abi4* and *cho1* mutants exhibit growth resistance to high concentrations of glucose. In contrast, CHO1, but not ABI4, is required for inhibition by excess of nitrate (Figure 1). The inhibitory action of different sugars on seed germination could proceed through different pathways, including complex interactions with those on phytohormone-response. A high level of glucose is responsible for the induction of ABA synthesis and also induces the expression of *ABI4* and *ABI5*. This suggests that at least some aspects of sugar signaling may be mediated by ABA response (Arroyo Becerra et al., 2001). Furthermore, glucose hypersensitivity results by overexpressing ABI3, ABI4, or ABI5, what suggests that these ABI genes could mediate the response to ABA and sugar (Finkelstein et al., 2002); in fact, the induction of these *ABI* genes is stronger when glucose is added to the medium during early stages of seedling development, right when the plant is most sensitive to the inhibitory effects of sugar (Gibson et al., 2001) and ABA induces ABI5 accumulation (Lopez-Molina et al., 2001). In *Lotus japonicus*, at concentrations up to 200 μM, nitrite and nitrate alleviated glucose-induced delay of seed germination, in a NO mediated response (Zhao et al., 2008).

Several sugars may delay seed germination via different pathways. Price et al. (2003) showed that glucose modulates a decreased in ABA concentration during germination in wildtype *Arabidopsis* seeds (Figure 1). Seed germination of *Lotus japonicus* was reported to be delayed by exogenous glucose (Zhao et al., 2008) and there was a significant inhibitory effect on seed germination with 2.5% glucose after 72 h. Inhibition of seed germination by glucose was substantially alleviated by exogenous supply of SNP (sodium nitroprusside), an exogenous donor of NO, (2.5% glucose + 200 μM SNP), and this effect was significantly reduced by cPTIO [carboxy-PTIO, 2-(4-carboxyphenyl)-4,4,5,5-tetramethylimidazoline-1-oxyl-3-oxide], a NO scavenger (200 μM), suggesting that the effect of SNP is likely due to NO. Germination of *Lotus japonicus* seeds was insensitive to KNO_2_ and KNO_3_ at concentrations up to 200 μM in the absence of glucose. At these concentrations, nitrite and nitrate alleviated glucose-induced delay of seed germination.

Auxins have been involved in cell-wall remodeling (Swarup et al., 2008), and imbibed after-ripened seeds, showed an enhanced expression of genes related to RNA translation, protein degradation and cell wall modification than dormant seeds (Holdsworth et al., 2007), and the expression of these genes increases within 1 and 3 h in imbibed after-ripened seeds (Nakabayashi et al., 2005). Genes involved in auxin transport (*AUX1*, *PIN1*, *PIN7*) are highly upregulated following imbibition of after-ripened seeds (Cadman et al., 2006; Carrera et al., 2007) or addition of gibberellin (GA; Ogawa et al., 2003), suggesting the initiation of root growth at the onset of phase III.

In short, seed germination is controlled by nutrient status through multiple action points. This implies a bona fide perception of nutrient balance, acting through signaling pathways either repressing or promoting seed germination.

### Root and Shoot Development

C-status ultimately controls many aspects of plant development. Vegetative growth is controlled by the cellular metabolic status (Lastdrager et al., 2014). Sugars are long-distance signals and there are sugar-dependent regulatory networks in roots. SnRK1 is a growth inhibitor under nutrient stress conditions (Guérinier et al., 2013). Sugar-phosphates regulate plant SnRK1 (Ghillebert et al., 2011). Glucose-6-phosphate (G6P), glucose-1-phosphate (G1P), and trehalose-6-phosphate (T6P) are repressors of SnRK1 activity (Zhang et al., 2009). Sucrose promotes the accumulation of T6P by inhibiting SnRK1 activity, thereby inducing biosynthetic processes and plant growth. Plant SnRK1 controls several important enzymes, such as nitrate reductase and sucrose phosphate synthase (Purcell et al., 1998). This suggests that SnRK1 could play a relevant role in controlling C/N metabolism (Coruzzi and Zhou, 2001). The ABA effect could be related to SnRK1, since phosphatases implicated in ABA signaling seem to inhibit SnRK1 activity through SnRK1 dephosphorylation (Rodrigues et al., 2013). *Arabidopsis* group C/S1 basic leucine zipper (bZIP) consists of nine bZIP transcription factors network and depends on the presence of bZIP proteins in a particular cellular context. The C/S1 network of bZIP factors works efficiently to integrate signals (Weltmeier et al., 2009). All these bZIP factors are expressed specifically in sink organs like young leaves, anthers and seeds. This fact suggests that the C/S1 network is involved in nutrients allocation to sink organs (Rook et al., 1998a,b). Sucrose inhibits the translation of S1-group bZIP mRNAs. The SnRK1 pathway has been assigned the function of adjusting the growth and development of the plant to its energy status (Baena-González et al., 2007). The SnRK1 pathway increases the transcriptional potential of S1 class bZIP proteins (Weltmeier et al., 2009) and SnRK1 seems to be implicated in the down-regulation of ribosomal protein (Baena-González et al., 2007). In a high metabolic status, T6p inhibits SnRK1, and the active TOR kinase stimulates translation and growth, being important for S1-group bZIP protein synthesis capacity via effects on ribosome biogenesis, mRNA polysome loading and mRNA translation (Rook et al., 1998a; Schepetilnikov et al., 2013).

Sucrose-induced repression of translation (SIRT) of the S1-group bZIP transcription factors has been confirmed for all five *Arabidopsis* S1-group members (Rook et al., 1998a; Wiese et al., 2005; Weltmeier et al., 2009). Sucrose stimulates the activation of the AKIN complex in *Arabidopsis* (Bhalerao et al., 1999) and plant SNF1 may mediate the activation of gene expression by sucrose and glucose (Purcell et al., 1998; Bhalerao et al., 1999). PRL1 is a potential subunit of the *Arabidopsis* AKIN complex and is a putative negative regulator of AKIN, yet the mutant is hypersensitive toward glucose (Nemeth et al., 1998). Two *Arabidopsis* protein kinases, KIN10 and KIN11, seem to be involved in the control of transcription convergent reprogramming as a response to darkness, sugar and stress conditions, three apparently unrelated factors; in addition, specific bZIP transcription factors partially mediate primary KIN10 signaling. KIN10 targets promote catabolism and suppress anabolism by sensing and signaling sugar and energy deprivation (Baena-González et al., 2007).

Hexokinases play an important role in a hexokinase-dependent sugar response pathway (Moore et al., 2003). Several glucose signal transduction pathways are known: a first one is AtHXK1-dependent, a second one is glycolysis-dependent and influenced by AtHXK1 activity, and a third pathway that is AtHXK1-independent (Xiao et al., 2000). The hexokinase-signaling pathway might play a role in cell-cycle control linked to the carbohydrate status, and sugar regulation of cycD2 seems to be mediated by hexokinase (Riou-Khamlichi et al., 2000). *Arabidopsis* HXK1 plays a relevant role in a number of glucose responses, namely cell proliferation, root and inflorescence growth, leaf expansion and senescence, and reproduction. HXK1 can play different functions in glucose signaling and metabolism; in fact, many of these new HXK1 functions can be performed at least partially by catalytically inactive HXK1 mutants (Moore et al., 2003). Growth promotion or inhibition by HXK1 would depend on glucose concentration, cell type, developmental state, and environmental condition (Moore et al., 2003). In the absence of nitrate, low glucose level signaling is HXK1 mediated but is independent of ABA and ethylene signaling. At low glucose levels, *ABI4* and *ABI5* gene expression is HXK1 dependent, while at high glucose levels these genes are expressed independently of HXK1 (Cho et al., 2010).

Sucrose effects on auxin levels are more pronounced in roots than in shoots, suggesting that sugars may impact auxin transport and/or conjugation pathways as well (Ljung et al., 2015). Auxins and CKs present links to sucrose sensing and signaling, and can function as short- and long-distance signaling molecules. They can play a role in integration of growth and development between shoot and root (Ljung et al., 2015). The rapid response around 30 min after adding 15 mM sucrose to C-starved seedlings, of an Aux/IAA family member IAA5 (Abel et al., 1995) is indicative of early crosstalk with auxins (Osuna et al., 2007). Auxin biosynthesis is induced by soluble sugars, and daily fluctuations in sugar content are correlated with fluctuations in auxin levels (Sairanen et al., 2012). In *Arabidopsis*, exogenous sucrose or glucose supply triggers an over-accumulation of auxin and increases the auxin flux in the hypocotyl notably through the up-regulation of the genes encoding auxin biosynthetic enzymes, including *CYP79B2/3* and *YUCCA8/9* (Lilley et al., 2012; Sairanen et al., 2012). Sucrose supplementation, required for rhythmic hypocotyl elongation, induces *YUCCA9* in shoots but not roots and sucrose effects on auxin levels are more pronounced in roots than in shoots, suggesting that sugars may impact auxin transport and/or conjugation pathways as well (Ljung et al., 2015). Auxins and sugars can be transported from shoot to root, inducing lateral root development in order to increase water and nutrients uptake from the soil, in turn increasing shoot growth capacity (Ljung et al., 2015).

CKs regulate the expression of sucrose transporters involved in phloem unloading adjusting sugar partitioning and sink strength, and they also regulate invertases and hexose transporters involved in sucrose catabolism and uptake in sink tissues (Roitsch and González, 2004; Proels and Roitsch, 2009).

**FIGURE 3:**
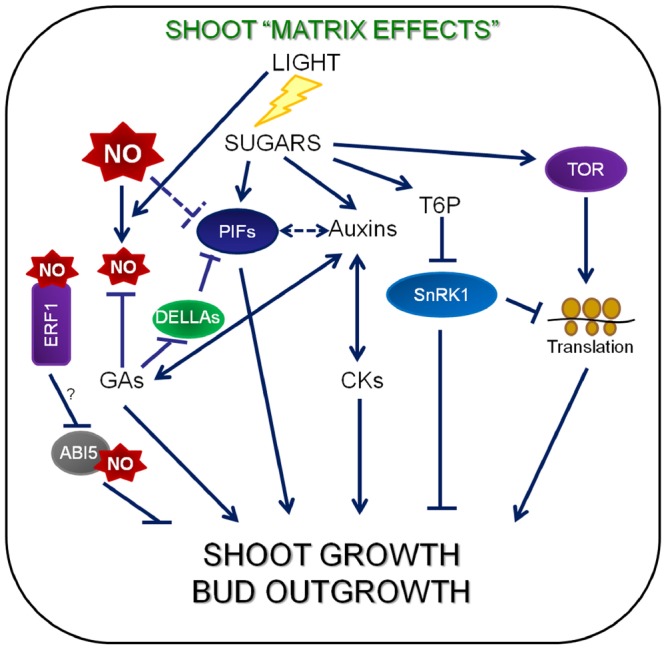
**Molecular components of sugar signaling networks and their involvement in shoot growth.** CKs, auxins and sugars function as long-distance signals. When photosynthesis takes place, sugars content increase in shoot and low sugars in roots implies that GAs are transported from the roots to the shoots and send sugars to the low sugar roots. Sucrose availability (high metabolic status) shows a good correlation with the level of plant T6P, which acts as an inhibitor of SnRK1. The active TOR kinase is important for C/S1-group bZIP protein synthesis and enhances translation and growth. Sucrose down-regulates the expression of C/S1-group *bZIP* at the translation level and the C/S1-bZIP transcription factor network is involved in the regulation of SnRK1 target genes (Lastdrager et al., 2014). The repression of ribosomal protein gene expression by SnRK1 (Baena-González et al., 2007) inhibits translation. Sucrose effects on auxin levels are more pronounced in roots than in shoots, suggesting sugars may impact auxin transport and/or conjugation pathways as well. DELLA and PIF proteins are good candidates for molecular hubs operating at the crossroads of many pathways (Ljung et al., 2015). There are NO and GA antagonist functions in the control of light-regulated photomorphogenesis through the balance between DELLAs and PIFs (Lozano-Juste and León, 2011). ERF1 has been suggested to function in light-regulated control of hypocotyl elongation, in a different way to the Cys-Arg/N-end rule pathway (Zhong et al., 2012).

Sugars like trehalose 6-phosphate (Tre6P) is both a signal of sucrose status and a negative feedback regulator of sucrose levels (Yadav et al., 2014; Figure 3). Tre6P can affect developmental processes such as shoot branching. The impact of sugar during the systemic regulation of bud outgrowth in response to either decapitation or light intensity has been analyzed by Barbier et al. (2015). Axillary buds outgrowth is driven by sugar availability independently of auxin levels. The combination of high sugar levels in shoot and high sugar sink strength in buds (high photosynthesis rate) drives to high branching. Sugars play a signaling role, and not only a trophic role (Barbier et al., 2015).

Slower growth at night in the starchless mutant, which shows higher sucrose levels during the day and absence of sugars at the end of the night (Wiese et al., 2007), requires an adjustment of GAs level to match the lower growth potential deriving from the lack of sugars at night (Paparelli et al., 2013). The dwarfism of GA-deficient mutants is, instead, uncoupled from carbon availability (Ribeiro et al., 2012), indicating that GA is primarily required for growth. DELLA proteins seem to represent a point of convergence for the hormonal and sucrose-dependent regulatory networks and DELLA are involved in the sucrose-GA interaction (Loreti et al., 2008; Figure 3). Sucrose-dependent stabilization of DELLAs (Li et al., 2014) seems to be a simple mechanism to connect sugars with other signaling pathways (Ljung et al., 2015). Cell expansion in the elongation zone of growing roots is controlled by GA, and the endodermis plays a key role in this process. Growth of these root tissues is regulated by endodermis-specific removal of DELLA repressor proteins mediated by GA, ensuring that cells from all elongation zones respond similarly to GAs expanding at the same rate (Ubeda-Tomás et al., 2008).

The Phytochrome-Interacting Factor (PIF) family of transcription factors seem to have their basic helix-loophelix in every process involving light, temperature and growth (De Lucas and Prat, 2014). DELLA and PIF proteins are good candidates for molecular hubs operating at the crossroads of many pathways (Ljung et al., 2015; Figure 3). PIFs attenuate the light signal through negative feedback on phytochrome transcription, as well as by bringing them along when they are targeted for proteasome-mediated degradation (Ni et al., 2014). A combination of circadian clock and light regulation control the activity of PIF4 and PIF5, leading to predictable daily oscillations in seedling growth rates (Nozue et al., 2007). These PIF-driven growth cycles depend on supplying seedlings with exogenous sucrose (Liu et al., 2011; Stewart et al., 2011; Figure 3).

Nitric oxide regulates GA signaling by controlling DELLA abundance and function. Lozano-Juste and León (2011) suggested that NO could induce the accumulation of DELLAs, despite finding no transcriptional induction of DELLA genes. There are NO and GA antagonist functions in the control of light-regulated photomorphogenesis through the balance between DELLAs and PIFs (Lozano-Juste and León, 2011; Figure 3).

A sugar-sensing pathway has been discovered in cultured *Arabidopsis* cells: protein phosphorylation, 14-3-3 binding, and regulatory proteolysis control diverse target proteins in C/N metabolism and signaling. Several cytosolic enzymes involved in C/N metabolism (nitrate reductase, glutamine synthetase, sucrose-phosphate synthase, trehalose-6-phosphate synthase and glutamyl tRNA synthetase) are relevant targets of this pathway (Cotelle et al., 2000; Finnemann and Schjoerring, 2000). We must consider the matrix effect, since phosphorylation and 14-3-3 binding of distinct subsets of these enzymes seem to be regulated by photosynthesis and hormones (Coruzzi and Zhou, 2001). When cells are starved of metabolizable sugars, 14-3-3-binding is lost and its targets are clipped by a specific cysteine protease.

Further studies are required to elucidate the signals responsible for nutrient signaling pathways in the regulation of root and shoot growth. CKs, auxins, GAs and sugars function as long-distance signals and a low level of sugar in roots implies that GAs are transported from the roots to the shoots. Sucrose availability, which implies a high metabolic status, is correlated with plant T6P levels and T6P inhibits SnRK1. It is known that metabolic events associated with a high concentration of carbohydrates could be crucial rather than the high concentrations of carbohydrate (Krapp et al., 1993). Additionally, the balance between DELLAs and PIFs is suggested to control light-regulated photomorphogenesis (Lozano-Juste and León, 2011).

## C and N Interaction Pathways (C/N Balance)

C/N signaling systems are influenced by the biological context such as cell-type, developmental, metabolic, and/or environmental conditions, as already suggested by Coruzzi and Zhou (2001).

As previously mentioned, only a few genes involved in C/N balance response have been described. NRT2.1 and NRT2.2 are the main highaffinity nitrate transporters in roots under N-replete conditions (Orsel et al., 2002). In their absence, an additional loss of function of NRT2.4 has a significant impact on plant fresh weight in N-starved plants suggesting a complex interaction between them in response to changes in N availability (Kiba et al., 2012). NRT2.1 is also known to play a role in the repression of lateral root initiation under high sucrose/low nitrate context, acting as either a sensor or a signal transduction protein in low nitrate conditions (Little et al., 2005). However, additional studies are required in order to elucidate how NRT2.1 regulates pericycle cell divisions in function of C/N external balance.

*GLR1.1* (putative glutamate receptor 1.1) functions as a molecular regulator in the high C/low N response and its consequent implication in ABA metabolism and sensitivity, and response to water stress in *Arabidopsis*. AtGLR1.1 is a component of the HXK1-independent ABA pathway in germinating seeds and functions as a sensor pointing its true ligand to the Glu. GLR1.1 can be expressed in the presence of a N source such as nitrate or amino acids (Glu or Gln), promoting seed germination (Kang and Turano, 2003; Kang et al., 2004).

*OSU1/QUA2/TSD2* encodes a putative methyltransferase which acts in cell wall biogenesis (Mouille et al., 2007) and is required for normal C/N nutrient balance response in plants (Gao et al., 2008). OSU1/QUA2/TSD2 might have different targets which in turn regulate pectin biosynthesis and C/N balance responses, respectively. This represents a surprising link between cell wall biogenesis and C/N balance response (Zheng, 2009). OSU1 is likely to act as a negative modulator either in distinct pathways leading to high C/N ratio or low C/N ratio responses or in a single pathway regulating various transcription factors in response to these distinct C/N imbalance conditions (Zheng, 2009).

Ubiquitin ligases are known to play an essential role in C/N balance, mediating nutrient-responsive pathways. KEEP ON GOING (KEG), a RING-type ubiquitin ligase in *Arabidopsis*, localizes to trans-Golgi network/early endosome (TGN/EE) vesicles and adjusts ABI5 levels by poly-ubiquitination in function of C and N metabolite availability (Stone et al., 2006). It has been proposed that KEG recognizes ABI5 through one or more of its ANK (Ankyrin) repeats (Stone et al., 2006) and its interaction with EDR1 is mediated by the HERC2-like repeats (Gu and Innes, 2011). KEG plays a relevant role in promoting post-germinative growth (Stone et al., 2006). Pointing in this direction is ATL31, a RING-H2-type ubiquitin ligase, which plays a key role in regulating the response to C/N conditions during post-germinative growth in *Arabidopsis*. Under low C/N conditions, ATL31 target protein degradation through 26s proteasome pathway entails post-germinative growth. The ATL31 protein localizes to the membrane and recruits target proteins for ubiquitination and degradation by the 26S proteasome, allowing seedling development proceed through the early post-germinative growth arrest checkpoint (Sato et al., 2009). Protein kinases catalyze ATL31 phosphorylation, which binds to 14-3-3 proteins and mediates their stability. In low C/high N conditions, the protein kinases catalyzing ATL31 phosphorylation are mostly inactivated. In high C/low N conditions, these protein kinases are active and catalyze the phosphorylation of 14-3-3 binding sites on ATL31, which binds 14-3-3 proteins and consequently perform their ubiquitination and proteasomal degradation (Yasuda et al., 2014). Studies carried out by Peng et al. (2007) demonstrated that *Arabidopsis* plants are equipped with a molecular mechanism to adapt to nitrate limitation in which NLA (Nitrogen Limitation Adaptation), a putative RING-type ubiquitin ligase, is involved in the ubiquitination-mediated degradation or modification of substrate protein(s) and function as a positive regulator for the adaptation response to nitrogen limitation.

There are recent experiments pointing out the involvement of specific ABA and SnRK1s signaling pathways in C/N response under ABI1 regulation. In high C/low N condition, Lu et al. (2015) identified genes whose expression is upregulated as *RD29b*, *LEA3-4*, and *TSPO* and suppressed in *ABI1* over-expressing plants, whereas other genes as *RAB18*, *AREB1*, and *ABF3*, were not affected. Additionally, SNRK1s-responsive genes as *DIN6* and *SEN5* were downregulated in wild-type plants and this effect was suppressed in *ABI1* over-expressing plants. SnRK1s proteins are known to phosphorylate several 14-3-3 targeting proteins, which play a role in C and N metabolism, such as nitrate reductase (NR) and sucrose 6-phosphate synthase (SPS; Comparot et al., 2003).

Carbon- and nitrogen-signaling pathways interact according to the external C/N status and plants have a C/N sensing and regulatory mechanism. Genome-wide analysis due to C and N signaling interactions in *Arabidopsis* revealed that C is a more ubiquitous regulator of the genome than N (Palenchar et al., 2004). These authors identified 3 MIPS FunCats corresponding to metabolism, protein synthesis and energy genes that are regulated by C/N signaling. The accurate regulation of metabolic genes is essential to cope with C/N status. The identification of putative *cis*-acting regulatory elements involved in C and N signaling interactions indicates the existence of multiple mechanisms for C/N status response. Two transcriptional mechanisms are suggested: (i) independent regulation of C-element and C-Nelement and (ii) dependent regulation of N-dependent enhancer of C regulation on a C-responsive transcription factor and *cis* element (Palenchar et al., 2004).

Taken together, a complex picture emerges in which nitrate transporters, glutamate receptors, methyltransferases and ubiquitin ligases are acting on multiple levels to integrate C and N signaling interaction pathways and regulate energy and metabolic genes as well as protein expression. An exquisite control comprises of several transcriptional mechanisms assuring an accurate response for the C/N status.

## Concluding Remarks

In this review, we have focused on how changes in C and N levels regulate the production of NO, which acts in plant developmental processes through the interaction with phytohormones and other plant growth regulators, using similar molecular elements. Recent investigations have the goal to shed light on the molecular mechanisms underlying the crosstalk of nutrients with NO and ABA response. This ambitious goal includes several tasks such as analyzing the regulation of NO and ABA production by changes in the C/N status, the characterization of mutants impaired in either NO or nutrients response (*cue1*, *Atnoa1-2*, *nia1nia2*, *gin5*, *gin6*…), the participation of nutrients in the regulation of lateral root growth by NO and, finally, the functional characterization of transcription factors responsive to nutrients, NO and ABA, unraveling their implication in different nutrients signaling pathways. NO is key to control many of the developmental outcomes in response to N, C and N/C. NO interference on acropetal auxin transport controls root development (Fernández-Marcos et al., 2011) and NO nitrosylates ABI5 as a regulator to control ABA hormone signaling through its degradation (Albertos et al., 2015). The implementation of the “omics” sciences will help to get insight into the nutrients and hormones crosstalk signaling pathways.

It is important to underline that our current understanding of C and N-dependent signaling pathways in seeds germination and plant development is mainly related to the model plant Arabidopsis. Therefore, it is necessary to elucidate nutrientsensing and signaling pathways in other plants.

As a useful application of the knowledge of these molecular mechanisms, development of perennial versions of important grain crops is crucial for the increasing worldwide food demand. Root branching order is the main determinant of root trait variation among species (Picon-Cochard et al., 2012) and nutrients can influence root structure. Perennial crops generally have advantages over annuals in maintaining important ecosystem functions, particularly on marginal landscapes or where resources are limited (Tilman et al., 2009). Therefore, identification and molecular characterization of genes involved in nutrients crosstalk with root development signaling pathways could be the basis for the generation of perennial crops through crop breeding.

### Conflict of Interest Statement

The authors declare that the research was conducted in the absence of any commercial or financial relationships that could be construed as a potential conflict of interest.
